# Correlated Response on Growth Traits and Their Variabilities to Selection for Ovulation Rate in Rabbits Using Genetic Trends and a Cryopreserved Control Population

**DOI:** 10.3390/ani11092591

**Published:** 2021-09-03

**Authors:** Rosa Peiró, Celia Quirino, Agustín Blasco, María Antonia Santacreu

**Affiliations:** 1 Centro de Conservación y Mejora de la Agrodiversidad Valenciana (COMAV), Universitat Politècnica de València (UPV), P.O. Box 22012, 46071 Valencia, Spain; ropeibar@btc.upv.es; 2 Laboratório de Reprodução e Melhoramento Genético Animal, Universidade Estadual do Norte Fluminense (UENF), Campos dos Goytacazes 28013-602, Brazil; crq@uenf.br; 3 Instituto de Ciencia y Tecnología Animal (ICTA), Universitat Politècnica de València (UPV), P.O. Box 22012, 46071 Valencia, Spain; ablasco@dca.upv.es

**Keywords:** control population, genetic parameters, growth rate, ovulation rate, slaughter weight, variability of growth traits, weaning weight

## Abstract

**Simple Summary:**

A successful response was obtained after selection for ovulation rate during 10 generations in rabbits. However, no correlated response in litter size was observed due to an increase in prenatal mortality. This increase could be due to the reduction in fetus weights and/or an increase in variable asynchrony among fetus weights. Therefore, the consequences of the selection procedure on weight at 28 and 63 days old (weaning and commercial time, respectively) and its variability are unknown. Using genetic trends and a cryopreserved control population for estimating correlated responses to selection, no relevant response on weight at 28 and 63 days old was observed. Similar results have been obtained for the variability of growth traits.

**Abstract:**

The aim of this work was to estimate correlated responses in growth traits and their variabilities in an experiment of selection for ovulation rate during 10 generations in rabbits. Individual weight at 28 days old (IW28, kg) and at 63 days old (IW63, kg) was analyzed, as well as individual growth rate (IGR = IW63 − IW28, kg). The variability of each growth trait was calculated as the absolute value of the difference between the individual value and the mean value of their litter. Data were analyzed using Bayesian methodology. The estimated heritabilities of IW28, IW63 and IGR were low, whereas negligible heritabilities were obtained for growth variability traits. The common litter effect was high for all growth traits, around 30% of the phenotypic variance, whereas low maternal effect for all growth traits was obtained. Low genetic correlations between ovulation rate and growth traits were found, and also between ovulation rate and the variability of growth traits. Therefore, genetic trends methods did not show correlated responses in growth traits. A similar result was also obtained using a cryopreserved control population.

## 1. Introduction

Intensive rabbit production is produced by a three-way cross in which males, selected for growth traits from paternal lines, are mated with crossbred females from lines selected for reproductive traits [1]. All these traits have economic importance [2] and it is important to know the genetic relationship between them. Crossbred females provide 50% of genes to terminal rabbits; therefore, maternal lines should also have an acceptable level for growth traits. On the other hand, paternal lines should also have an adequate level for reproductive traits (including litter size and ovulation rate) to ensure line maintenance and selection through time.

Selection for ovulation rate, proposed as a way to increase litter size [3], has been successful to increase the number of ova shed, but no correlated response on litter size has been observed in the unique experiment performed in rabbits [4]. However, the correlated response on growth traits is unknown. In this experiment, females belonging to the line selected for increase ovulation rate had more implanted embryos according to the increase in ovulation rate [5,6]. An increment in the number of ova shed and implanted embryos has also been observed in maternal commercial rabbit lines selected for higher litter size [7,8]. Some authors show how higher competition for space and nutrition during the fetal period, after implantation, could reduce fetal weight [9,10] and modify its variability [10]. Maternal and litter effects during gestation could have a relevant effect on young rabbits after birth in the growth period due to an increase in ovulation rate. A reduced fetal weight might lead to lower weight at birth and subsequently affect weaning and commercial weight [11].

The objective of this study was to estimate the correlated response on growth traits and their variability using genetic trends methods and also by comparing the selected line with a cryopreserved control in a rabbit line selected for ovulation rate during 10 generations. Maternal and litter effects on these traits were also evaluated.

## 2. Materials and Methods

### 2.1. Ethical Statement

All experimental procedures were approved by the Universitat Politècnica de València Research Ethics Committee, according to Council Directives 98/58/EC and 2010/63/EU.

### 2.2. Animals and Selection Procedure

The animals used in the experiment came from a synthetic line selected for the phenotypic value of ovulation rate in the second gestation of each female during 10 generations. The selection procedure has been described previously by Laborda and coauthors [4].

All animals were kept under constant photoperiod of 16 h light:8 h dark and controlled ventilation. The female nourished its kits during lactation period without fostering. Young rabbits for all parities were weaned at 28 days old and placed in flat-deck cages, 8 rabbits per cage, and fed ad libitum with a commercial diet (crude protein, 16.1%; crude fiber, 16.5%; ether extract, 4.4%; ash, 8.1% as-fed basis; NANTA S.A.; Valencia, Spain). The fattening period was 35 days. At 63 days old, rabbits were slaughtered or selected and placed in individual flat-deck cages and fed ad libitum with a commercial diet (crude protein, 17.5%; crude fiber, 15.5%; ether extract, 5.4%; ash, 8.1% as-fed basis; NANTA S.A.; Valencia, Spain) and they stayed there up to their reproductive age: 17–18 weeks of age.

To produce a control population, embryos from 45 donor females and 10 males from the base generation were vitrified and stored in liquid nitrogen until they were transferred. Details of the procedure are presented by Laborda and coauthors [6]. The offspring of the cryopreserved animals were used in order to avoid the effect of recovery, cryopreservation and transferring techniques on growth characteristics suggested by Cifre and coauthors [12]. The offspring of the control population was contemporary to animals from the 10th generation.

### 2.3. Traits

The reproductive trait analyzed during the second gestation in the selection procedure during ten generations was ovulation rate (OR), measured as the number of corpora lutea. The OR was also recorded in the last gestation of all females, that did not die or were not culled, obtaining a total of 1478 records from 856 females. A total of 20,230 records were used to analyze individual weight at 28 days old (IW28, kg) and 19,362 records were used to analyze individual weight at 63 days old (IW63, kg) and individual growth rate (IGR = IW63 − IW28, kg). An approach to study variability of growth traits was performed using deviation of growth traits, which was estimated as the absolute value of the difference between the individual value for IW28, IW63 and IGR and the mean value of their litter, DIW28, DIW63 and DIGR in kg, respectively.

All growth traits were also measured in the offspring of the control population and in contemporary animals from the 10th generation, obtaining a total of 1238 and 1142 records for IW28 and 1208 and 1123 records for IW63 and IGR, respectively.

### 2.4. Statistical Analyses

All analyses were performed using Bayesian methodology [13,14].

Correlated responses to selection have been estimated using two independent approaches: genetic trends and control population. Genetic trends use all data from all generations of selection but its estimations depend on the model, and therefore response to selection depends on the genetic parameters used in the model. In the control population approach, the number of data is smaller but differences between control and selected population will have only genetic bases if both populations are raised contemporarily.

#### 2.4.1. Genetic Parameters and Genetic Trends

A repeatability model was used to analyze OR in the selection period:

y_ijklm_ = YS_i_ + PO_j_ + L_k_ + a_ijkl_ + p_ijkl_ + e_ijklm_ (1)

where y_ijklm_ is the trait OR; YS_i_ is the effect of year-season of the mating day (31 levels for OR); PO_j_ is the effect of the parity order (four levels for OR); L_k_ is the effect of lactation status at mating (two levels: lactating and nonlactating does when mated); a_ijkl_ is the additive value of the animal; p_ijkl_ is the permanent environmental effect; and e_ijklm_ is the residual effect.

For individual weights and IGR, as well as for growth variability traits, the animal model used was:

y_ijklmn_ = YS_i_ + PO_j_ + NBA_k_ + d_ijkl_ + c_ijklm_ + a_ijklmn_ + e_ijklmn_ (2)

where y_ijklmn_ is IW28, IW63 and IGR; YS_i_ is the fixed effect of year-season in which the animal was growing (29 levels); PO_j_ is the effect parity in which the animal was born (five levels); NBA_k_ is the effect of the number of rabbits born alive when the animal was born (17 levels); d_ijkl_ is the random dam (or female) effect between parities (851 levels); c_ijklm_ is the common litter effect (2683 levels); a_ijklmn_ is the additive value of animal; and e_ijklmn_ is the residual effect.

Univariate analysis for OR was performed to estimate the heritability of the selection trait. To account for the selection process and to estimate the heritability of each growth trait as well as the correlation between OR and growth traits, bivariate analyses including OR were performed.

Data augmentation [14,15] was performed to analyze the data in order to have the same design matrices for all traits since different models for OR and growth traits were used. Augmented data are not used for inferences but allow the simplification of computing.

After data augmentation, the model for all traits was:

(3) $$\left(y|b,a,p,d,c,R_{0}\right)∼N\left(Xb+Za+Wp+Dd+Cc,R_{0}⊗I_{n}\right)$$

where y is a vector of augmented data; X, Z, W, D and C are known incidence matrices; and R is the (co)variance residual matrix. Records of different individuals were assumed to be conditionally independent, given the parameters, but a correlation between residuals of different traits of the same individual was allowed.

Hence, sorting the data by individual, the residual (co)variance matrix can be written as $R_{0}⊗I_{n}$, with $R_{0}$ being the 2×2 residual (co)variance matrix between OR and the growth trait analyzed and $I_{n}$ being an identity matrix of the same order as the number of individuals. Bounded uniform priors were used to represent vague previous knowledge of environmental effects, b. Prior knowledge concerning the other random effects was represented by assuming that they were normally distributed, conditionally on the associated variance components. Thus, for the additive genetic effects:

(4) a|G∼N(0,G)

where 0 is a vector of zeroes and G is the genetic variance covariance matrix. Sorting the data by individual as before, this matrix can be written as $G_{0}⊗A$, where $G_{0}$ is the 2×2 genetic (co)variance matrix between traits analyzed and A is the known additive genetic relationship matrix between elements of the additive genetic effects vector.

The distribution of permanent environmental effects was assumed to be normal and of the form:

(5) p|P∼N(0,P)

where 0 is a vector of zeroes and P is the permanent effect matrix. Sorting the data by individual, this matrix can be written as $P_{0}⊗I_{p}$, with $P_{0}$ being the 2×2 permanent variance matrix between traits analyzed and $I_{p}$ being the identity matrix with the same order of the number of levels of permanent effects.

The distribution of the random dam effects between parities was assumed to be normal and of the form:

(6) d|D∼N(0,D)

where 0 is a vector of zeroes and D is the random dam effects between parities matrix. Sorting the data by individual, this matrix can be written as $D_{0}⊗I_{d}$, with $D_{0}$ being the 2×2 permanent variance matrix between traits analyzed and $I_{d}$ being the identity matrix with the same order of the number of levels of the random dam effects between parities.

Finally, the distribution of the common litter effects was assumed to be normal and of the form:

(7) c|C∼N(0,C)

where 0 is a vector of zeroes and C is the common litter effects matrix. Sorting the data by individual, this matrix can be written as $C_{0}⊗I_{c}$, with $C_{0}$ being the 2×2 common litter variance matrix between traits analyzed and $I_{c}$ being the identity matrix with the same order of the number of levels of the common litter effects.

For all analyses, bounded flat priors were used for matrices $R_{0}$, $G_{0}$, $P_{0}$, $D_{0}$ and $C_{0}$.

To estimate the correlations between growth traits, trivariate analyses including OR and two growth traits were performed. Hence, sorting the data by individual, the residual (co)variance matrix can be written as $R_{0}⊗I_{n}$, with $R_{0}$ being the 3×3 residual (co)variance matrix between OR and the growth traits analyzed and $I_{n}$ being an identity matrix of the same order as the number of individuals. As previous analyses, bounded uniform priors were used for environmental effects and normally distributed priors, conditionally on the associated variance components that were used for random effects. The additive genetic effects, the permanent environmental effects, the random female effects between parities and the common litter effects were the same as described above. As in the previous model, all effects are independent among them. However, sorting the data by individual as before, the G matrix can be written $G_{0}⊗A$, where $G_{0}$ is the 3×3 genetic (co)variance matrix between traits analyzed and A is the known additive genetic relationship matrix described previously; the P matrix can be written as $P_{0}⊗I_{p}$, with $P_{0}$ being the 3×3 permanent variance matrix between traits analyzed and $I_{p}$ being the identity matrix with the same order of the number of levels of permanent effects; the D matrix can be written as $D_{0}⊗I_{d}$, with $D_{0}$ being the 3×3 permanent variance matrix between traits analyzed and $I_{d}$ being the identity matrix with the same order of the number of levels of the random dam effects between parities; and the C matrix can be written as $C_{0}⊗I_{c}$, with $C_{0}$ being the 3×3 permanent (co)variance matrix between traits analyzed and $I_{c}$ being the identity matrix with the same order of the number of levels of the common litter effects.

Marginal posterior distributions of all unknowns were estimated using a Gibbs sampling procedure using the program TM [16]. Different confidence intervals were estimated: k_95%_ is the guaranteed value of the interval [k, 1] containing the 95% of the probability, P is the probability of the estimation being higher (or lower) than 0.00, P_0.10_ and P_0.30_ are the probability of the estimation being higher (or lower) than 0.10 and 0.30, respectively, and P_r_ is the probability of relevance [13,14]. We considered a heritability to be irrelevant when it was lower than 0.10 [4,5,6,17]. In the case of correlation, we considered to be an irrelevant value all correlations in absolute value lower than 0.30, since the percentage of the variance explained by the other trait (r^2^) is <10%. After some exploratory analyses, two chains were used, each of 1,000,000 iterations, with a burning period of 200,000 iterations. Only every 100th iteration was saved. Features of marginal posterior distributions of parameters were obtained using the package R code. Convergence was tested using the Z criterion of Geweke and Monte Carlo sampling errors were computed.

#### 2.4.2. Selected versus Control Population

The model assumed for analyzing OR using the offspring of the control and selected population was:

y_ijklmn_ = Line_i_ + YS_j_ + PO_k_ + L_l_ + p_ijklm_ + e_ijklmn_ (8)

where y_ijklm_ is the trait OR; Line_i_ is the effect of the line (two levels: control and selected); YS_j_ is the effect of year-season (three levels); PO_k_ is the effect of parity (two levels: at second and fourth gestation); L_l_ is the effect of lactation state of the doe (two levels: lactating and nonlactating does when mated); p_ijklm_ is the effect of the doe (105 levels); and e_ijklmn_ is the residual of the model.

The model assumed for analyzing individual growth traits and their variabilities was:

y_ijklmn_ = YS_i_ + PO_j_ + NBA_k_ + d_ijkl_ + c_ijklm_ + e_ijklmn_ (9)

where y_ijklm_ is IW28, IW63 and IGR; YS_i_ is the fixed effect of year-season in which the animal was growing (four levels); PO_j_ is the effect parity in which the animal was born (four levels); NBA_k_ is the effect of the number of rabbits born alive when the animal was born (16 levels); d_ijkl_ is the random dam effect between parities (96 levels); c_ijklm_ is the common litter effect (302 levels); and e_ijklmn_ is the residual effect.

Bounded uniform priors were used for all unknowns with the exception of the dam and common litter effects, which were considered normally distributed. Dam effect was with mean 0 and variance $I\sigma_{d}^{2}$, where ***I*** is a unity matrix and $\sigma_{d}^{2}$ is the dam effect variance of the trait. Common effect was with mean 0 and variance $I\sigma_{c}^{2}$, where ***I*** is a unity matrix and $\sigma_{c}^{2}$ is the common effect variance of the trait. Residuals were normally distributed with mean 0 and variance $I\sigma_{e}^{2}$. The priors for the variances were also bounded uniform positive. Features of the marginal posterior distribution of differences between line means were estimated by using the Gibbs sampling algorithm. Similarly to previously described genetic estimations, chains of 1,000,000 samples each were used, with a burning period of 200,000. One sample out of each 100 was saved to avoid high correlations between consecutive samples. Features of marginal posterior distributions of differences between line means were obtained using the package R code. Convergence was tested using the Z criterion of Geweke and Monte Carlo sampling errors were computed.

## 3. Results

### 3.1. Genetic Parameters and Genetic Trends

Descriptive statistics for all traits are presented in Table 1. Rabbits at 28 and 63 days old weighed 0.52 and 1.76 kg, respectively. The coefficient of variation of growth traits was close to 0.20, whereas their variability, estimated as the absolute value of the difference between the individual value and the mean value of their litter, was close to 0.90.

Phenotypic correlations between OR and the growth traits were low: 0.17, 0.15 and 0.09 for IW28, IW63 and IGR, respectively. The R-squared between OR and the other analyzed traits was lower than 5%. When the ratio between R-squared and R-squared maximum was estimated, different results were observed: 0.72 for IW28, 0.57 for IW63 and 0.29 for IGR.

Phenotypic residual correlations between OR and growth traits and also between OR and the variability of growth traits were very low (Table 2). Only correlations between weights (IW28–IW63) and between IGR and IW63 were higher than 0.60. Low correlations were also found within the variability of growth traits and also between growth traits and their variabilities. These results showed that the accuracy was low when the model included some effects to correct the data.

The heritability for OR was moderate, 0.17, with a probability to be higher than 0.10 of 94% and the highest posterior density region at 95% ranging from 0.08 to 0.27 (data not shown). The heritability values of IW28, IW63 and IGR were low: 0.09, 0.12 and 0.11, respectively (Table 3). A heritability higher than 0.10 was considered relevant for growth traits and their variability. The probability of the heritability being higher than 0.10 was high for IW63 and IGR. The maternal effects of the doe over all their parities, which is calculated as the ratio of the maternal effect variance with respect to phenotypic variance (m^2^), had relevance for IW28 (0.14), and it was close to zero for IW63 and IGR. However, the common litter effect, which is calculated as the ratio of the common litter effect variance with respect to phenotypic variance (c^2^), explained a greater part of phenotypic variance for all growth traits, with their values being close to 0.30. Genetic variation for the variability of growth traits was negligible, lower than 0.02, similarly to the maternal effects. The common litter effects for the variability of growth traits were close to 0.10.

All high posterior density regions for genetic correlations between OR and growth traits were large (Table 4). The estimate of the genetic correlation between OR and IW28 was very low (0.11), whereas between OR and IW63 and between OR and IGR it was 0.23 and 0.28, respectively. The assumed relevant value for correlation was 0.30 (in absolute value). The probability that the genetic correlation between OR and growth traits was higher than 0.30 was lower than 50%. Moderate positive genetic correlation between OR and variability of growth traits was obtained, although a low accuracy was achieved since the highest posterior density region at 95% was wide.

The genetic correlation between IW28 and IW63 was positive (P > 0 = 100%) and high (mean = 0.83) (Table 5). Moreover, the probability that the genetic correlation was at least 0.71 was 95%. A similar result was obtained when the genetic correlation between IW63 and IGR was analyzed; the mean was 0.95 and the probability that the genetic correlation was at least 0.92 was 95%. Moreover, genetic correlations between DIW63 and the other analyzed variability of growth traits were also higher than 0.90. However, non-relevant genetic correlations between growth traits and their variabilities were observed, since the probability of relevance is lower than 0.30.

**Figure 1:**
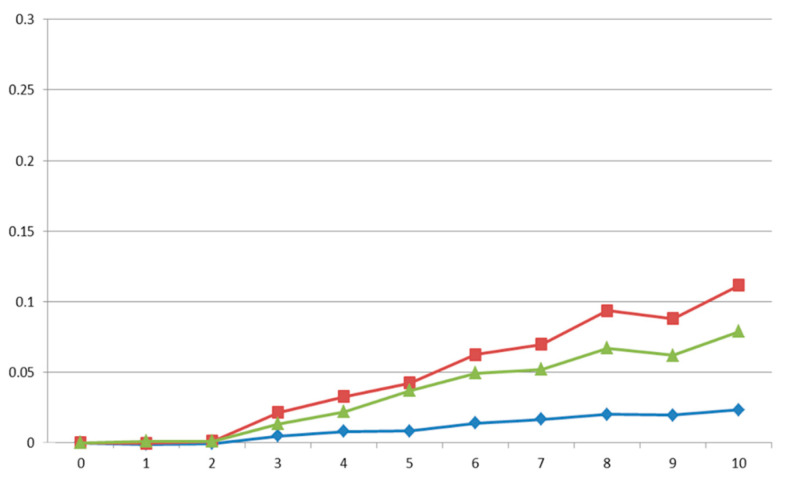
Genetic trends for individual weight at 28 days old (blue line; kg), individual weight at 63 days old (red line; kg) and individual growth rate (green line; kg).

**Figure 2:**
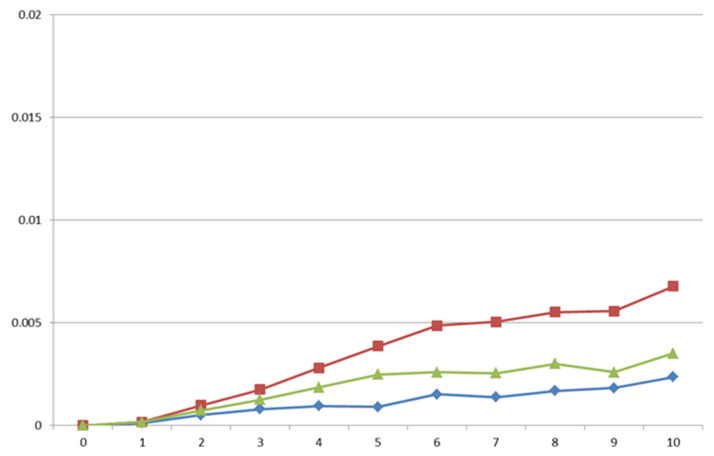
Genetic trends for variability of individual weight at 28 days old (estimated as the absolute value of the difference between IW28 and the mean value of their litter at 28 days old; blue line; kg), variability of individual weight at 63 days old (estimated as the absolute value of the difference between IW63 and the mean value of their litter at 63 days old; red line; kg) and variability of individual growth rate (estimated as the absolute value of the difference between IGR and the mean value of their litter for growth rate; green line; kg) of the OR line.

The correlated responses to selection were estimated at the end of the selection period as the difference of the average breeding values between the end and the beginning of the period. Correlated responses on IW28, IW63 and IW2863 were low (Figure 1), being 2.3, 11.2 and 7.9 g per generation, respectively. These correlated responses corresponded to 0.4, 0.6 and 0.6% per generation, respectively. However, the correlated response on the variability of growth traits was close to zero (Figure 2).

### 3.2. Control Population

Table 6 shows raw means and coefficients of variation for the growth traits measured in the control population. Rabbits at 28 and 63 days old belonging to the control population weighed 0.50 and 1.77 kg, respectively. The coefficient of variation of growth traits was smaller than the coefficient of variation of the variability of growth traits.

Features of the marginal posterior distributions of the differences between the control and selected populations for growth traits and their variabilities are presented in Table 7. A relevant response to selection (R value) was assumed when the difference between populations was at least 10% of the mean of the control population, corresponding to an increase of 1% per generation. Growth traits presented no relevant differences between the control and selected populations (0.02, 0.07 and 0.08 for IW28, IW63 and IW2863, respectively). For these traits, the probability of relevance was lower than 15%. Similarly, no correlated responses on the variability of growth traits were observed.

Similarly to the estimations using genetic trends, the common litter effect, c^2^, explained a greater part of the phenotypic variance for growth traits, close to 0.25, whereas low estimations were observed for the variability of growth traits. The maternal effect of the doe over all their parities, m^2^, had a non-negligible value for IW28 (0.10).

## 4. Discussion

Direct response to selection for ovulation rate was obtained in rabbits after 10 generations of selection, 0.13 ova per generation. However, prenatal survival decreased and no correlated response on litter size at birth was observed [4]. The decrease in prenatal survival can be explained by a higher fetal competition due to an increment of implanted embryos [5]. Higher competition among fetuses for space, nutrients and blood supply in the uterus reduces birth weight [18]. This has led to the question of whether selection for ovulation rate could reduce weight at birth and subsequently weaning weight (IW28), commercial weight (IW63) and individual growth rate (IGR) [11], as well as modify their variability (DIW28, DIW63 and DIGR, respectively). On the other hand, in pigs, a line selected for ovulation rate with no response in litter size showed a high response in litter size when directly selected for litter size [19]; thus, a line selected for ovulation rate can be interesting for further research to improve litter size.

Descriptive statistics obtained in the present work are similar to those previously reported in other rabbit maternal lines [8,17,20] at the same ages. Phenotypic correlations between ovulation rate and growth traits were low, similarly to correlation between ovulation rate and the variability of growth traits. As expected, weaning and commercial weight were highly related and commercial weight and growth rate were also highly associated, in agreement with previous publications in rabbits [8,17]. Weights at birth were not available but high correlations between weight at birth and weight at weaning have been found by Argente and coauthors [18]. Finally, low phenotypic correlations were also found between growth traits and the variability of growth traits. To our knowledge, these are the first phenotypic estimations between growth traits and their variability.

### 4.1. Genetic Parameters

Low heritabilities, close to 0.10, were obtained for IW28 and IW63, which corresponded to weaning and commercial time, respectively. Likewise, IGR, which is the most common selection criterion in sire lines in rabbits [1], also showed a low heritability. Similar heritabilities for these three traits were obtained by Peiró and coauthors [17], who analyzed a line selected by ovulation rate and litter size, and also for IW28 by Mínguez and coauthors [20], who analyzed four maternal lines (A, V, H and LP). However, a broad range of heritabilities, ranging from 0.03 to 0.25, has been obtained for these traits in paternal rabbit lines (reviewed by Garcia and coauthor) [21]. The common litter effect is more important than the maternal effect and additive genetic effect in all growth traits, close to 0.3 for growth traits and to 0.1 for the variability of growth traits. However, low maternal effects have been obtained for all growth traits, in agreement with previous results [8,17,20,21]. Regarding the variability of growth traits, negligible estimations (P > 0.10 = 0.00) were observed for heritabilities and the maternal effect and low values were obtained for the common litter effect, around 0.10, as it is mentioned earlier. Estimations were in the range of those published previously by Peiró and coauthors [17] in a maternal line. The preweaning environment effect, common litter effect and maternal effect have a large influence on studied growth traits, although there is a reduction in the preweaning environment effect over time, as previously found by other authors [8,17,20]. This reduction should be related to kits’ separation from their mothers and also for kits’ distribution into different cages (it is common to separate the litter into several cages, so litters from different rabbit does can be mixed).

Genetic and phenotypic correlations were similar, and this is a generally observed phenomenon [22]. Genetic correlations between ovulation rate and growth traits and their variability were similar to the only estimation found in the literature [17]. Genetic correlation between growth traits was positive and high in agreement with [23,24,25]. Genetic correlation between commercial weight (IW63) and IGR was higher than between IW28 and IGR. These results were previously observed by McNitt and coauthor [23] and by Ezzeroug and coauthors [25]. Similar behavior has been observed for genetic correlations between the variability of growth traits; a higher genetic correlation was observed when DIW63 was evaluated, close to 0.90. Moderate–high genetic correlations, close to 0.60, were observed between the variability of individual weight at 28 days old and the variability of the individual growth rate. However, no relevant genetic correlations between each growth trait and its variability have been obtained. To our knowledge, there is no information about genetic correlations between the variability of growth traits.

### 4.2. Correlated Response

Considering all these genetic parameters, the correlated response to selection for ovulation rate during ten generations was estimated at the end of the selection period. Using genetic trends methods, no relevant correlated responses on IW28, IW63 and IW2863 were found, less than 1% of the trait per generation. These estimates agreed with previous results [17]. Similarly, no relevant correlated responses on the variability of growth traits were observed, less than 1% of the trait per generation. Using the comparison of the selected line with a cryopreserved control population method, no correlated response was observed. The use of a cryopreserved control population corroborates the results obtained with genetic trends, and neither correlated response to selection for ovulation rate during 10 generations on individual weight at 28 and 63 days old nor on their variability was observed. As the response to selection estimated by control and selected populations is the same as the one obtained using the model by genetic trends that includes maternal and common litter effects, we can infer that dam effects were not modified after selection.

## 5. Conclusions

The increase in ovulation rate by selection did not reduce the weight of the young rabbits at weaning (28 days) and at marketing (63 days) and did not modify their variabilities. It can be inferred that female effects, which include maternal and common litter effect, were not modified after selection.

## Figures and Tables

**Table 1:** Raw means, coefficients of variation (CV) and number of data (N) for growth traits and their variability.

	Mean	CV	N
IW28	0.52	0.23	20,230
IW63	1.76	0.14	19,362
IGR	1.24	0.14	19,362
DIW28	0.05	0.85	20,230
DIW63	0.11	0.91	19,362
DIGR	0.09	0.99	19,362

IW28: individual weight at 28 days old (kg), IW63: individual weight at 63 days old (kg), IGR: individual growth rate (kg), DIW28: variability of individual weight at 28 days old (estimated as the absolute value of the difference between IW28 and the mean value of their litter at 28 days old; kg), DIW63: variability of individual weight at 63 days old (estimated as the absolute value of the difference between IW63 and the mean value of their litter at 63 days old; kg), DIGR: variability of individual growth rate (estimated as the absolute value of the difference between IGR and the mean value of their litter for growth rate; kg).

**Table 2:** Phenotypic residual correlations between ovulation rate (OR, ova) and growth traits (kg) and their variabilities (estimated as the absolute value of the difference between the individual value and the mean value of their litter; kg).

	OR	IW28	IW63	IGR	DIW28	DIW63
IW28	0.11					
IW63	0.08	0.64				
IGR	0.03	0.35	0.90			
DIW28	0.05	0.12	0.09	0.13		
DIW63	0.04	0.11	0.10	0.11	0.21	
DIGR	0.06	0.09	0.09	0.12	0.15	0.35

IW28: individual weight at 28 days old, IW63: individual weight at 63 days old, IGR: individual growth rate, DIW28: variability of individual weight at 28 days old, DIW63: variability of individual weight at 63 days old, DIGR: variability of individual growth rate.

**Table 3:** Features of marginal posterior distributions of the heritability (h^2^), ratio of the maternal effect variance with respect to phenotypic variance (m^2^) and ratio of the common litter effect variance with respect to phenotypic variance (c^2^) for growth traits and their variability.

h^2^					m^2^		c^2^	
Trait	Mean	HPD_95%_	k_95%_	P_0.10_	Mean	k_95%_	Mean	k_95%_
IW28	0.09	0.04, 0.15	0.05	0.37	0.14	0.17	0.33	0.35
IW63	0.12	0.07, 0.19	0.08	0.79	0.04	0.06	0.29	0.31
IGR	0.11	0.06, 0.17	0.07	0.71	0.01	0.02	0.30	0.32
DIW28	0.01	0.00, 0.02	0.01	0.00	0.01	0.00	0.08	0.10
DIW63	0.00	0.00, 0.01	0.00	0.00	0.01	0.00	0.10	0.13
DIGR	0.01	0.00, 0.02	0.01	0.00	0.01	0.00	0.13	0.15

HPD_95%_: highest posterior density region at 95%, k_95%_: limit of the interval [k, 1] containing a probability of 95%, P_0.10_: probability of the proportion being higher than 0.10, IW28: individual weight at 28 days old, IW63: individual weight at 63 days old, IGR: individual growth rate, DIW28: variability of individual weight at 28 days old (estimated as the absolute value of the difference between IW28 and the mean value of their litter at 28 days old), DIW63: variability of individual weight at 63 days old (estimated as the absolute value of the difference between IW63 and the mean value of their litter at 63 days old), DIGR: variability of individual growth rate (estimated as the absolute value of the difference between IGR and the mean value of their litter for growth rate).

**Table 4:** Features of the estimated marginal posterior distributions of the genetic correlations be-tween ovulation rate (OR) and growth traits and their variability.

Trait	Mean	HPD_95%_	P	P_0.30_
OR-IW28	0.11	−0.26, 0.50	0.71	0.21
OR-IW63	0.23	−0.13, 0.56	0.90	0.39
OR-IGR	0.28	−0.12, 0.63	0.93	0.48
OR-DIW28	0.28	0.02, 0.59	1.00	0.56
OR-DIW63	0.62	0.40, 0.79	1.00	1.00
OR-DIGR	0.55	0.17, 0.76	1.00	0.54

HPD_95%_: highest posterior density region at 95%, k_95%_: limit of the interval [k, 1] containing a probability of 95%, P: probability of the correlation being higher than zero, P_0.30_: probability of the absolute value of correlation being higher than 0.30, IW28: individual weight at 28 days old, IW63: individual weight at 63 days old, IGR: individual growth rate, DIW28: variability of individual weight at 28 days old (estimated as the absolute value of the difference between IW28 and the mean value of their litter at 28 days old), DIW63: variability of individual weight at 63 days old (estimated as the absolute value of the difference between IW63 and the mean value of their litter at 63 days old), DIGR: variability of individual growth rate (estimated as the absolute value of the difference between IGR and the mean value of their litter for growth rate).

**Table 5:** Features of the estimated marginal posterior distributions of the genetic (r_g_) and common litter (r_c_) correlations between individual weights.

	r_g_					r_c_	
Traits	Mean	HPD_95%_	k_95%_	P	P_0.30_	Mean	k_95%_
IW28-IW63	0.83	0.70, 0.93	0.71	1.00	1.00	0.69	0.73
IW28-IGR	0.40	0.07, 0.69	0.11	0.99	0.64	0.15	0.21
IW63-IGR	0.95	0.91, 0.98	0.92	1.00	1.00	0.91	0.93
DIW28-DIW63	0.93	0.79, 0.99	0.82	1.00	1.00	0.53	0.57
DIW28-DIGR	0.62	0.33, 0.87	0.38	1.00	1.00	0.29	0.32
DIW63-DIGR	0.95	0.86, 0.99	0.87	1.00	1.00	0.80	0.86
IW28-DIW28	0.18	−0.02, 0.44	0.00	0.97	0.26	0.04	0.08
IW63-DIW63	−0.18	−0.54, 0.18	−0.50	0.58	0.29	−0.20	−0.15
IGR-DIGR	−0.21	−0.48, 0.09	−0.45	0.57	0.28	−0.23	−0.20

HPD_95%_: highest posterior density region at 95%, k_95%_: limit of the interval [k, 1] containing a probability of 95%, P: probability of the r_g_ being higher than zero when the mean is positive or lower than zero when it is negative, P_0.30_: probability of the r_g_ being higher than 0.30 when the mean is positive or lower than −0.30 when the mean is negative, IW28: individual weight at 28 days old, IW63: individual weight at 63 days old, IGR: individual growth rate, DIW28: variability of individual weight at 28 days old (estimated as the absolute value of the difference between IW28 and the mean value of their litter at 28 days old), DIW63: variability of individual weight at 63 days old (estimated as the absolute value of the difference between IW63 and the mean value of their litter at 63 days old), DIGR: variability of individual growth rate (estimated as the absolute value of the difference between IGR and the mean value of their litter for growth rate).

**Table 6:** Raw means, coefficients of variation (CV) and number of data (N) for growth traits and their variability in the offspring of the control population.

	Mean	CV	N
IW28	0.50	0.25	1238
IW63	1.77	0.14	1208
IGR	1.26	0.15	1208
DIW28	0.05	0.81	1238
DIW63	0.10	0.90	1208
DIGR	0.06	0.87	1208

IW28: individual weight at 28 days old (kg), IW63: individual weight at 63 days old (kg), IGR: individual growth rate (kg), DIW28: variability of individual weight at 28 days old (estimated as the absolute value of the difference between IW28 and the mean value of their litter at 28 days old; kg), DIW63: variability of individual weight at 63 days old (estimated as the absolute value of the difference between IW63 and the mean value of their litter at 63 days old; kg), DIGR: variability of individual growth rate (estimated as the absolute value of the difference between IGR and the mean value of their litter for growth rate; kg).

**Table 7:** Features of the estimated marginal posterior distributions of the differences between the control and selected population for growth traits and their variability.

	Control–Selected	HPD_95%_	P	R	P_R_
IW28	0.02	−0.01, 0.06	0.89	0.05	0.12
IW63	0.07	0.01, 0.15	0.98	0.17	0.00
IGR	0.08	0.04, 0.13	1.00	0.13	0.02
DIW28	0.001	−0.001, 0.003	0.75	0.005	0.00
DIW63	0.001	0.000, 0.002	0.76	0.001	0.42
DIGR	0.003	0.002, 0.004	1.00	0.006	0.01

HPD_95%_: highest posterior density region at 95%, P: probability of the difference between the control and selected population being higher than zero, R = 10% of the mean of the control population (1% per generation), P_R_ = probability of response, which is the probability of the difference being higher than R, IW28: individual weight at 28 days old, IW63: individual weight at 63 days old, IGR: individual growth rate, DIW28: variability of individual weight at 28 days old (estimated as the absolute value of the difference between IW28 and the mean value of their litter at 28 days old), DIW63: variability of individual weight at 63 days old (estimated as the absolute value of the difference between IW63 and the mean value of their litter at 63 days old), DIGR: variability of individual growth rate (estimated as the absolute value of the difference between IGR and the mean value of their litter for growth rate).
